# Synaptic and memory dysfunction induced by tau oligomers is rescued by up-regulation of the nitric oxide cascade

**DOI:** 10.1186/s13024-019-0326-4

**Published:** 2019-06-27

**Authors:** Erica Acquarone, Elentina K. Argyrousi, Manon van den Berg, Walter Gulisano, Mauro Fà, Agnieszka Staniszewski, Elisa Calcagno, Elisa Zuccarello, Luciano D’Adamio, Shi-Xian Deng, Daniela Puzzo, Ottavio Arancio, Jole Fiorito

**Affiliations:** 1 Taub Institute for Research on Alzheimer’s Disease and the Aging Brain, 630 West 168th Street, P&S 12-420D, New York, NY 10032 USA; 2 DiMi Department of Internal Medicine and Medical Specialties, University of Genoa, 16132 Genoa, Italy; 3 Faculty of Psychology and Neuroscience, Maastricht University, 6229 Maastricht, Netherlands; 4 Department of Biomedical and Biotechnological Sciences, Section of Physiology, University of Catania, 95125 Catania, Italy; 5 Department of Experimental Medicine, Section of General Pathology, School of Medical and Pharmaceutical Sciences, University of Genoa, 16132 Genoa, Italy; 6 Department of Pharmacology, Physiology and Neuroscience, Rutgers University, Newark, NJ USA; 7 Department of Medicine, Columbia University, New York, NY 10032 USA; 8 Oasi Research Institute-IRCCS, 94018 Troina, Italy; 9 Department of Pathology and Cell Biology, Columbia University, New York, NY 10032 USA; 10 Department of Life Sciences, New York Institute of Technology, Northern Boulevard P.O. Box 8000, Theobald Science Center, room 425, Old Westbury, NY 11568 USA

**Keywords:** Tau oligomers, Nitric oxide, Soluble guanylyl cyclase, PDE5, Protein kinase G, CREB, Memory, Alzheimer’s disease

## Abstract

**Background:**

Soluble aggregates of oligomeric forms of tau protein (oTau) have been associated with impairment of synaptic plasticity and memory in Alzheimer’s disease. However, the molecular mechanisms underlying the synaptic and memory dysfunction induced by elevation of oTau are still unknown.

**Methods:**

This work used a combination of biochemical, electrophysiological and behavioral techniques. Biochemical methods included analysis of phosphorylation of the cAMP-responsive element binding (CREB) protein, a transcriptional factor involved in memory, histone acetylation, and expression immediate early genes c-Fos and Arc. Electrophysiological methods included assessment of long-term potentiation (LTP), a type of synaptic plasticity thought to underlie memory formation. Behavioral studies investigated both short-term spatial memory and associative memory. These phenomena were examined following oTau elevation.

**Results:**

Levels of phospho-CREB, histone 3 acetylation at lysine 27, and immediate early genes c-Fos and Arc, were found to be reduced after oTau elevation during memory formation. These findings led us to explore whether up-regulation of various components of the nitric oxide (NO) signaling pathway impinging onto CREB is capable of rescuing oTau-induced impairment of plasticity, memory, and CREB phosphorylation. The increase of NO levels protected against oTau-induced impairment of LTP through activation of soluble guanylyl cyclase. Similarly, the elevation of cGMP levels and stimulation of the cGMP-dependent protein kinases (PKG) re-established normal LTP after exposure to oTau. Pharmacological inhibition of cGMP degradation through inhibition of phosphodiesterase 5 (PDE5), rescued oTau-induced LTP reduction. These findings could be extrapolated to memory because PKG activation and PDE5 inhibition rescued oTau-induced memory impairment. Finally, PDE5 inhibition re-established normal elevation of CREB phosphorylation and cGMP levels after memory induction in the presence of oTau.

**Conclusions:**

Up-regulation of CREB activation through agents acting on the NO cascade might be beneficial against tau-induced synaptic and memory dysfunctions.

**Electronic supplementary material:**

The online version of this article (10.1186/s13024-019-0326-4) contains supplementary material, which is available to authorized users.

## Background

An increased interest in Alzheimer’s disease (AD) research is now directed towards tau protein, a hallmark of the disease. Insoluble aggregates of tau are responsible for the formation of neurofibrillary tangles (NFTs). However, growing evidence is pointing at very soluble small tau aggregates in the etiopathogenesis of the disease, as they emerge as more acutely toxic than large insoluble aggregates. Extracellular oligomeric forms of tau (oTau) have been shown to affect memory and its cellular correlate, long-term potentiation (LTP) [1, 2]. However, despite the strong correlation between oTau and AD pathology [3, 4], the molecular mechanisms by which tau protein induces synaptic dysfunction and memory impairment remain unidentified.

There is a broad consensus that cyclic adenosine monophosphate (cAMP) responsive element binding (CREB) protein plays a crucial role in memory consolidation. CREB phosphorylation is a post-translational modification involved in gene transcription mechanisms leading to synaptic plasticity and memory formation (for a review see [5]) and is likely to be affected in AD [6–23]. CREB is at the crossroads of several molecular pathways and mechanisms that have been proposed as potential therapeutic targets against AD, including the nitric oxide (NO)/cyclic guanosine monophosphate (cGMP) dependent protein kinases (PKG)/CREB pathway, the cAMP-dependent protein kinases (PKA)/CREB pathway and the mitogen-activated protein kinase/extracellular regulated kinase (MAPK/ERK) pathway [16]. To this regard, the NO/cGMP/PKG/CREB cascade is particularly attractive because drugs boosting it, especially phosphodiesterase 5 (PDE5) inhibitors, are widely used for the therapy of erectile dysfunction and pulmonary hypertension [24], and it is therefore plausible that their administration is compatible with therapeutic usage.

NO, a gaseous molecule produced by the NO-synthase enzyme is involved in various steps of brain physiology, from development to synaptic plasticity and memory [25–27]. NO activates soluble guanylyl cyclase (sGC) which, in turn, produces cGMP [28], a cyclic nucleotide whose levels are also down-regulated by PDE5, an enzyme that specifically hydrolyzes the nucleotide. Following its production, cGMP activates PKGs, a family of kinases that have been implicated in the modulation of neurotransmission, LTP, and memory [29–32] and are capable of phosphorylating CREB. Intriguingly, proteomic and metabolomic studies have revealed disrupted NO homeostasis in AD [33]. Moreover, up-regulation of the NO cascade through drugs acting on its various molecular components has provided favorable results in studies aimed at finding strategies to counteract the damage of synaptic plasticity and memory by oligomers of Aβ [34–36], another toxic protein in AD. Given that oTau share a common molecular mechanism with Aβ oligomers when they impair memory and LTP in mice [37], we investigated whether the oTau-induced damage of synaptic function and memory can be rescued via up-regulation of the NO cascade.

## Methods

### Animals

All the experiments were performed using 3–4 month-old male and female C57BL/6 mice hosted at the Columbia University animal facility. The mice were maintained on a 12 h light/dark cycle in stable conditions in terms of temperature, humidity, and ventilation. Water and food were offered ad libitum*.*

### Preparation of recombinant tau

The tau 4R/2 N construct was prepared in expression vector pET29a (Bioclone; San Diego, CA, USA) in the bacterial strain BL21 (DE3) for protein expression, as previously described [1, 38]. Cells were streaked on LB agar ampicillin plates and a single colony was picked and grown overnight in a mixture of overexpression and expansion broth (Zymo Research; Irvine, CA, USA). Cells were pelleted by centrifugation at 6000 g for 30 min in a GS3 rotator at 4 °C and lysed in a 2% Triton-X-100 phosphate-buffered saline with a protein inhibitor mixture. Streptomycin sulfate was added to precipitate DNA. After centrifugation, the supernatant was heated at 100 °C for 15 min and the precipitate was removed by centrifugation at 15000 g for 20 min at 4 °C. After adding TCEP and 1% PCA, the pH of the supernatant was neutralized by using 1 N NaOH. For the first purification step, the supernatant was transferred to a slide-A-lyser cassette (30 K MWCO) and buffered exchanged to remove excess chemicals. Afterwards, the supernatant was loaded on His-Spin Protein Miniprep columns (Zymo Research; Irvine, CA, USA) and eluted with phosphate buffer containing 250 mM imidazole. For oligomerization, tau was transferred to a slide-A-lyser cassette and buffer exchanged with oligomerization buffer following incubation with 1 mM H_2_O_2_ at room temperature for 20 h for introducing disulfide bonds. Tau protein concentration was determined from the absorption at 280 nm with an extinction coefficient of 7450 cm-1 M-1 and oligomers were visualized through Western blotting. Oligomers were transferred in Tris-Acetate gels and then immunoblotted on nitrocellulose membrane. The primary antibody was diluted to a final concentration of 1:1000 for immunoblotting (anti-tau antibody; EP2456Y; Cambridge, United Kingdom). The secondary goat anti-rabbit antibody (1:10,000) was purchased from Thermo Fisher Scientific (Waltham, MA). In separate experiments, to rule out the possibility of memory defects due to the presence of high amounts of endotoxin released by bacteria necessary for recombinant Tau generation, we measured endotoxin levels in the oTau preparation using the HEK-Blue™ LPS Detection Kit2 (Invivogen). Measurement of the endotoxin concentration in 3 different batches of recombinant protein showed values equal to 1.2 ± 0.67 EU/ml (corresponding to ~ 0.24 × 10^− 6^ μg/μl). These values were similar to the endotoxin concentration observed in artificial cerebrospinal fluid (ACSF), which was equal to 1.2 ± 0.57 EU/ml (*n* = 3). Interestingly, the concentration of endotoxin affecting memory in rats after intracerebroventricular injection has been shown to be equal to 2.5 μg/μl [39]. Thus, the endotoxin concentration in the oTau preparation is not only similar to that observed in ACSF, but is also ~ 1 × 10^7^ lower than that affecting memory in rats, indicating that endotoxin cannot be held responsible for the observed effects of the oTau onto memory.

### Western blotting on mouse hippocampi

Mice were sacrificed through cervical dislocation 1 min or 60 min after a single 0.8 mA foot shock provided during the last 2 s at the end of a 30 s tone (2880 Hz at 85 Db) or sham exposure in a fear conditioning chamber. Hippocampi were separated from the rest of the brain and lysates were prepared as previously described [8]. Briefly, hippocampal tissue was homogenized in lysis buffer (62.5 mM Tris-HCl pH 6.8, 3% SDS, Halt™ Protease and Phosphatase Inhibitor Cocktail (100X) and incubated at 4 °C for 60 min, then sonicated before centrifugation at 13,000 rpm for 15 min. p-CREB antibodies (1:1000, 5% BSA in TRIS-buffered saline (TBS) 1X and Tween80), t-CREB antibodies (1:500, 5% milk in TBS), and GAPDH antibodies (1:5000, 5% milk in TBS) were from MilliporeSigma (St. Louis, MO, USA). Acetyl-histone 3 lysine 27 (acH3K27) antibodies (1:2000, 5% milk in TBS) and total histone H3 antibodies (1:5000, 5% milk in TBS) were from New England BioLabs (Ipswich, MA, USA). β-III-Tubulin antibodies (1:1000, 5% milk in TBS) were from Promega (Madison, WI, USA). Arc antibodies (1:1000, 5% BSA in TBS 1x and Tween80) and c-Fos antibodies (1:200, 5% BSA in TBS 1x and Tween80) were from Abcam (Cambridge, United Kingdom). For quantitative immunoblot analysis, equal amounts of proteins were loaded into each lane. Blots were re-probed with corresponding pan-antibodies and antibody for tubulin or GAPDH to confirm equal loading. For quantification, we used a signal in the linear range. Immunoblot data were quantified by measuring the band intensity using imaging software (NIH ImageJ).

### cGMP measurement in mouse hippocampi

Levels of cGMP were quantitated in duplicate by the Enzyme Immunoassay procedure (Cayman Chemical Company; Ann Arbor, MI, USA) following the manufacturer’s guidelines. Hippocampal lysates were harvested, immediately frozen in dry ice and weighted. Samples were homogenized in a 5% trichloroacetic acid solution containing 3-isobutyl-1-methylxanthine (100 μM) using a pellet pestle motor homogenizer and centrifuged at 6000 g for 20 min. The supernatant was collected, treated with a saturated ether solution, and heated for 20 min to remove the ether residual. cGMP was measured using a microplate reader (Tecan 200) using a wavelength of 410 nm.

### Drug preparation

For the LTP experiment, all the compounds were dissolved in ACSF to achieve the final concentration required. DEA/NO, ODQ, BAY41–2272, and 8-Br-cGMP were diluted in 0.1% DMSO; sildenafil and compound 7a in 0.05% DMSO. DEA/NO was stored for 24 h in alkaline solution (0.01 m NaOH) and diluted in ACSF immediately before use. Different treatments were interleaved on slices from the same mice. For the behavioral experiments, compound 7a and 8-pCPT-cGMP were dissolved in 2% DMSO and 2% Tween 80. Compound 7a was synthesized in six steps [35], while DEA/NO and BAY41–2272 were purchased from Enzo life Science (Farmingdale, NY, USA), 8-Br-cGMP from Biolog Life Science Institute (Bremen, Germany), ODQ from Cayman Chemical Company (Ann Arbor, MI, USA), sildenafil and 8-pCPT-cGMP from MilliporeSigma (St. Louis, MO, USA). For biochemical experiments compound 7a was dissolved in 4% DMSO and 2% Tween 80.

### Electrophysiological recordings

Mice were sacrificed through cervical dislocation and hippocampus was removed immediately after decapitation. Transverse hippocampal slices (400 μm) were cut on a tissue chopper and transferred to the recording chamber where the physiological conditions in the brain were maintained by perfusion of ACSF continuously bubbled with 95% O_2_ and 5% CO_2_. The ACSF consisted of (in mM): NaCl (124.0), KCl (4.4), Na_2_HPO_4_ (1.0), NaHCO_3_ (25.0), CaCl_2_ (2.0), MgCl_2_ (2.0), and glucose (10.0). Slices were allowed to recover for at least 90 min before commencing the extracellular field recordings. A bipolar tungsten electrode and a glass electrode filled with ACSF were placed in the Schaeffer collateral fibers and the CA1 *stratum radiatum*, respectively. An input-output analysis was utilized to determine the maximal slope and the baseline was recorded every minute at approximately 35% of the maximum evoked slope [40]. After establishing a 30 min stable baseline, LTP was induced using a theta-burst stimulation (four pulses at 100 Hz, with the bursts repeated at 5 Hz and each tetanus including three 10-burst trains separated by 15 s) and was recorded for 2 h after tetanization. LTP was measured as field-EPSP (fEPSP) slope expressed as a percentage of the baseline and the results were represented as mean ± SEM. For all the electrophysiological experiments, oTau and the various drugs were diluted in the ACSF and provided through the bath solution.

### Stereotaxic surgery and infusion method

Before fixing the mice in the stereotaxic device, anesthesia was induced by intraperitoneal (i.p.) injection with Avertin (500 mg/Kg). Animals were injected with analgesic (Carprofen subcutaneously on the back, 5 mg/Kg) and local anesthetic (Marcaine subcutaneously under the scalp 3 mg/Kg). A midline incision was made in the skull and the underlying area was cleared of tissue by using H_2_O_2_. The coordinates of the dorsal hippocampus were 2.46 mm posteriorly and 1.5 mm laterally from Bregma to a depth of 1.30 mm [41]. A 26-gauge guide cannula (PlasticsOne; Roanoke, VA) was fixed to the skull using acrylic dental cement (Paladur). After 6–9 days of recuperation period, awake mice were restrained and injected onto both dorsal hippocampi with oTau (500 nM, 1 μl per side, at 180 and 20 min prior to the task) or vehicle. For the injections, we utilized Hamilton syringes connected with a polyethylene tube at the end of which an internal microsyringe was fixed. The microsyringe was custom-made to reach a depth of 1.5 mm, ensuring that it was not extending beyond the cannula. Following injection, the microsyringe was left in place for 1 min on each side to ensure perfusion of the hippocampus with oTau. Correct positioning of the cannulas was verified at the end of the experiment by intrahippocampal injection of methylene blue.

### Behavioral studies

#### Spatial short-term memory

The RAWM consists of a white circular pool, 120 cm in diameter, filled with non-toxic white paint to make the water opaque. Within the pool there is an apparatus consisting of six arms radiating from the central area, forming six arms. Spatial cues were present on the walls of the room. Throughout the test, the water temperature was maintained stable at 24 ± 2 °C. The platform was positioned at the end of one of the arms, submerged in the water. The location of the platform (10 cm diameter) was kept constant for each mouse, while the starting position differed between the trials. The test took place for two consecutive days, and each mouse underwent 15 trials per day. On the first day, mice were trained for 15 trials, with the first 12 trials alternating between visible (platform flagged) and hidden (platform 1 cm beneath the water surface). The last 3 trials of the first day and all the 15 trials of the second day were done with hidden platform. In each trial, the mouse was allowed to swim freely for 60 s in the maze to find the platform. Once on the platform, the mouse was allowed to rest for 20 s and to observe the visual cues. If a mouse was unable to find the platform within 60 s, the experimenter guides it towards the platform for the 20 s stay. During the 1 min trial, each time the mouse entered an arm other than the goal arm (in which the platform was located), or if the mouse did not take any decision regarding which arm to explore within 10 s, an error was registered. Entry into an arm was defined as the entry of all the four paws of the mouse into the particular arm. After completing each trial, the mouse was removed from the pool, gently towel dried and placed back into its cage under a heat lamp. To avoid the learning limitations imposed by over practice and to prevent fatigue that may result from consecutive trials spaced practice training was established by running the mice in cohorts of 4 or 5 and alternating different cohorts through the 15 trials over the testing period each day. The result is shown by dividing the 30 trials into 10 blocks. Each block represents the error average of 3 consecutive trials. For these experiments, oTau was injected into dorsal hippocampi 180 and 20 min prior to starting the task both on the first and second day of RAWM testing, as described in the stereotaxic surgery and infusion method above. Compound 7a or 8-pCPT-cGMP, in turn, were administered i.p. after the 2nd, 4th, 7th and 9th block of trials.

#### Visible platform testing

The test has been utilized for the assessment of visual and/ or motor, and/or motivational deficits [36]. It was performed in the same pool as the RAWM. The test takes place in 2 consecutive days and mice underwent 2 sets of trials each day. Every set consisted of 3 trials in which the mouse trained to find the visible escaping platform, flagged with a bottle cup on the top. During one set of trials the platform was located in one of the three quadrants of the pool. The starting position of the mouse was kept constant for a specific location of the platform. The mice were placed gently on the water, facing the walls, and each trial lasted until the mouse had found the platform until the maximum time of 60 s. After the end of the trials mice were guided to the platform and allowed to observe environmental cues for 20 s. Time between entering the pool and reaching the platform (latency) and velocity were analyzed by using a video tracking system (Ethovision XT). The results were shown in 4 blocks and each block represents the average of one set of experiment. oTau was infused both days 180 and 20 min prior to the task, as described in the stereotaxic surgery and infusion method above. Treatment with either 7a or 8-pCPT-cGMP was administered after the first set of three trials each day.

#### Fear conditioning

The fear conditioning test was used for evaluating associative fear memory in rodents. The test consists in total of 3 days, in which the first day the animals are placed in the fear conditioning chamber (Noldus) for 2 min before the presentation of the conditional stimulus (tone; 2880 Hz at 85 Db). In the last 2 s of the tone, mice received the unconditional stimulus (foot shock; 0.8 mA). After the pairing of the 2 stimuli, mice were left in the chamber for another 30 s in the absence of a stimulus. The second day, mice were returned to the same conditioning chamber for another 5 min without the presence of tone of shock. Freezing behavior, distinguished by the absence of movement except breathing, was monitored during the test using a vision tracking and analysis system (Ethovision XT, Noldus). The third day, the cued fear memory was evaluated. For that, mice were placed in the same chamber with modified walls, floor, and vanilla odor, which represents a novel context. In the course of 5 min, 2 min of freely exploration was followed by exposure for 3 min in the conditional stimulus. Administration of oTau, 7a, or 8-pCPT-cGMP occurred on the first experimental day. Mice were injected with oTau 180 and 20 min prior to the test, whereas the two compounds were administered once immediately after the foot shock. The rationale of giving the drugs after the foot shock was to examine their effects in the consolidation phase of contextual fear memory. Additionally, administration of the drugs after the foot shock excludes the possibility of interference with the perception of the pain.

#### Open field

The test has been used for assessing exploratory behavior and anxiety levels [42]. Mice were placed in a novel open environment consisting of Plexiglass transparent walls (model ENV- 520; Med Associates, St. Albans, Vermont) (43.2 cm long × 43.2 cm width × 30.5 cm high). Mice were placed in the open field. Their activity was automatically recorded for 10 min, on two consecutive days. oTau was infused both days 180 and 20 min before the test, as described in the stereotaxic surgery and infusion method above. Additionally, mice were treated with either 7a or 8-pCPT-cGMP both days with a single injection immediately after the end of the test.

#### Sensory threshold assessment

The test was used for evaluating the animal perception of the shock. The test was performed the last day of experiments, and the animals were placed in the same chamber that the fear conditioning test took place. Animals subjected to 1 s foot shocks of increasing intensity from 0.1 to 0.7 mA at 0.1 mA increments every 30 s. Behavior was recorded by video capture software (Ethovision XT) and was evaluated manually. The graphs represent the average of the foot shock intensity that elicited the first visible response (flinching), the second motor response (jumping), and the first audible response (vocalization). Mice were injected with oTau 180 and 20 min prior to the task, as described in the stereotaxic surgery and infusion method above.

### Statistical analysis

For electrophysiological recordings, results were analyzed by ANOVA for repeated measures comparing traces after tetanic stimulation with treatment condition as the main effect. The average of the last 5 points of the curve was compared by using one-way ANOVA with Bonferroni’s post-hoc corrections. For the behavioral tests, animals were run in cohorts in which sex of mice was kept balanced across groups. Results were analyzed with ANOVA for repeated measures (RAWM errors and latency) or one-way ANOVA with Bonferroni post-hoc correction. For western blotting, conditions were compared by using unpaired t-test or one-way ANOVA. Statistical analysis was performed by using Systat 9 software (Chicago, IL, USA). Differences were considered significant at a *p* value less than 0.05. Results were expressed as Standard Error of the Mean (SEM).

## Results

### oTau affects molecular mechanisms underlying memory formation

**Fig. 1:**
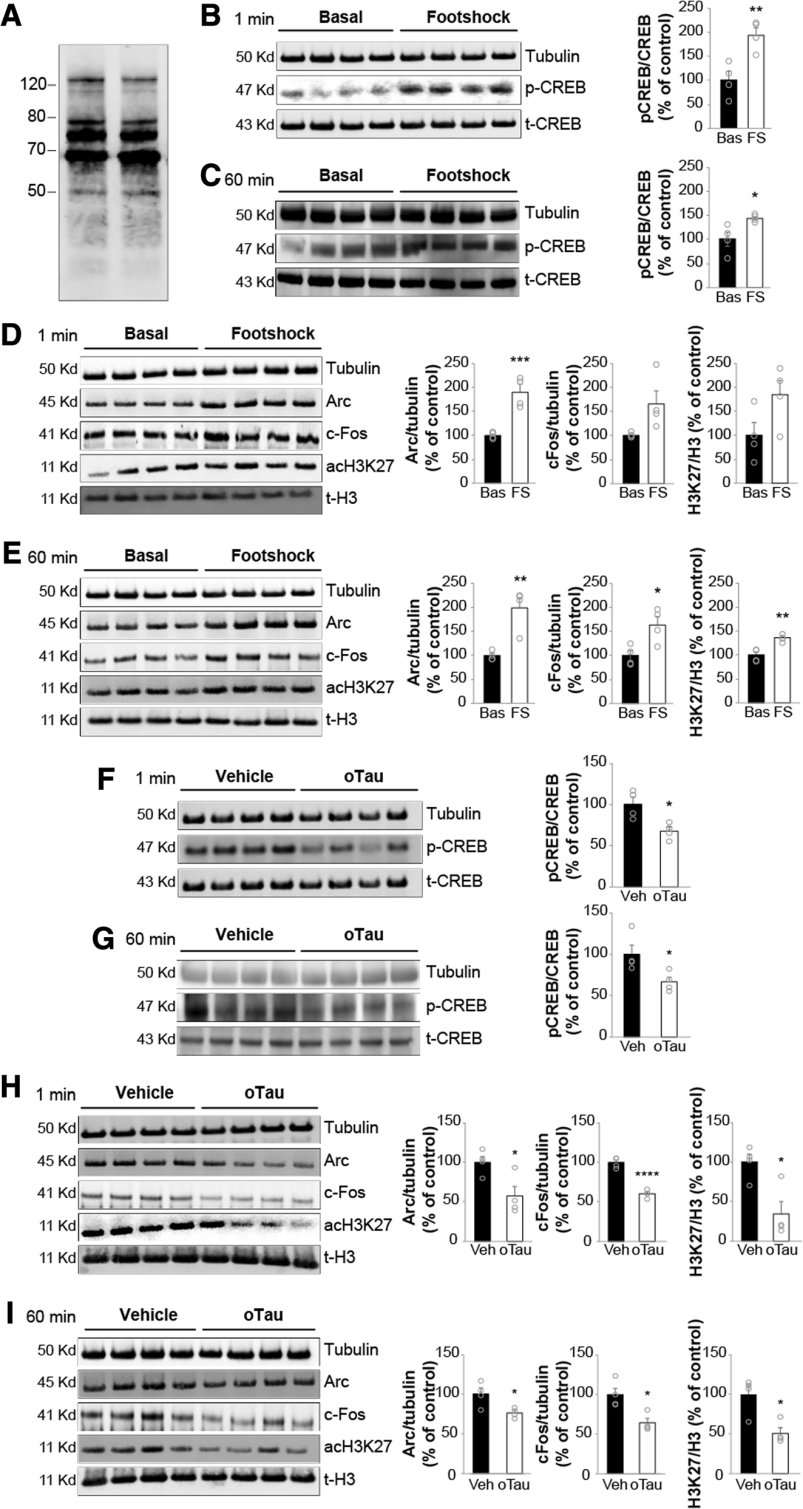
oTau impairs CREB phosphorylation during memory formation. **a** SDS-PAGE gel of recombinant oTau 4R/2 N preparation shows the presence of monomers and different size oligomers. **b-c** Phospho-CREB immunoblots of hippocampal homogenates from mice previously treated with/without a foot-shock to induce fear memory. Hippocampi were harvested 1 min (**b**) and 60 min (**c**) after the foot-shock or sham exposure in the fear-conditioning chamber. Graphs showing the average ratio of phospho-CREB (p-CREB)/total-CREB (t-CREB) (t-test at 1 min: *p* = 0.007; at 60 min: *p* = 0.03; *n* = 4 per each group, 2 males and females for each experimental condition; Bas = Baseline; FS = Footshock). **d-e** acH3K27, c-Fos and Arc immunoblots of hippocampal homogenates from mice under the same conditions as B-C. Graphs showing the average ratio of acH3K27/total-H3 (t-H3) (at 1 min: *p* = 0.08; at 60 min: *p* = 0.002), c-Fos (at 1 min: *p* = 0.05; at 60 min: *p* = 0.02), and Arc (at 1 min: *p* = 0.001; at 60 min: *p* = 0.004); n = 4 animals, 2 males and females for each experimental condition). **f-g** Phospho-CREB immunoblots of hippocampal homogenates from mice treated with vehicle (Veh) or oTau (22.95 μg/ml). Hippocampi were harvested 1 min (**f**) and 60 min (**g**) after the foot-shock. Graphs showing the average ratio of p-CREB/t-CREB (unpaired t-test at 1 min: *p* = 0.016; at 60 min: *p* = 0.044; n = 4 per each group, 2 males and females for each experimental condition). **h-i** acH3K27, c-Fos and Arc immunoblots of hippocampal homogenates from mice treated with vehicle (Veh) or oTau (22.95 μg/ml) under the same conditions as **f**-**g**. Graphs showing the average ratio of acH3K27/t-H3 (at 1 min: *p* = 0.014; at 60 min: *p* = 0.012), c-Fos (at 1 min: *p* < 0.0001; at 60 min: *p* = 0.013), and Arc (at 1 min: *p* = 0.025; at 60 min: *p* = 0.026). n = 4 animals, 2 males and females for each experimental condition. *p < 0.05, **p < 0.01; ***p < 0.005; ****p < 0.0001

Considering the profound effect that oTau exposure has on synaptic plasticity and memory [1, 2], we decided to determine whether the molecular mechanisms underlying memory formation, including CREB phosphorylation, are affected by oTau. As previously shown [1], different aggregation forms of Tau were present in our preparation (Fig. 1a). Foot-shock, a stimulus that is normally used for training in fear conditioning tests, increased phosphorylation of the memory-related molecule CREB both at 1 min and 60 min after the memory induction without affecting tubulin levels (Fig. 1b-c). This observation was associated with changes in the gene transcription machinery. Specifically, we detected a slight increase in acetylation of the chromatin-associated protein histone 3 (H3) at the lysine residue 27 (acH3K27), 1 min after the shock, which became significant at 60 min, as well as in the expression of the immediate early gene *Arc* that is implicated in memory formation, both at 1 and 60 min after the shock (Fig. 1d-e). Moreover, expression of another immediately early gene, involved in memory formation, *c-Fos*, was slightly increased at 1 min after the electric shock and more markedly at 60 min (Fig. 1d-e). Two intra-hippocampal injections of oTau (at 180 and 20 min prior to applying the electric shock) at a concentration of 22.95 μg/ml, previously found to impair memory formation [1], reduced the levels of phosphorylated CREB (pCREB), acH3K27, c-Fos, and Arc compared to vehicle-treated control mice after both 1 min and 60 min without affecting tubulin levels (Fig. 1f-i). oTau did not produce a modification of pCREB, acH3K27, c-Fos, and Arc without foot-shock stimulation (data not shown). Collectively, these data demonstrated that similar to extracellular Aβ oligomers [7], molecular mechanisms involved in memory formation are inhibited by extracellular oTau.

### Increase in NO levels through the NO donor, DEA/NO, protects against oTau-induced impairment of LTP

**Fig. 2:**
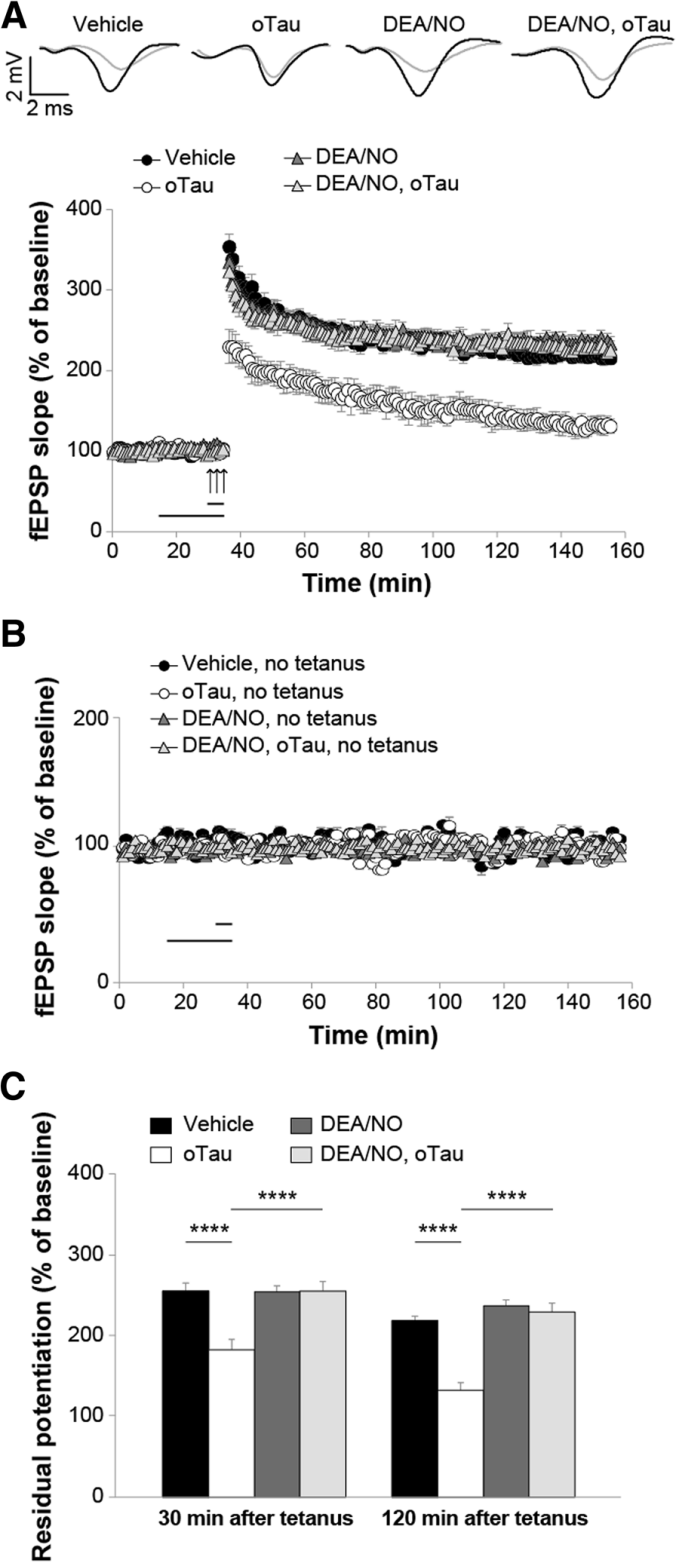
oTau induced LTP impairment was rescued by the NO donor DEA/NO. **a** LTP was impaired in hippocampal slices from mice perfused for 20 min before tetanus with 100 nM oTau compared to vehicle-treated slices (ANOVA for repeated measures: F_(1,19)_ = 30.859, p < 0.0001). A perfusion with DEA/NO (3 μM, 5 min) rescued the LTP impairment in slices concomitantly treated with oTau (100 nM, 20 min) in experiments that were interleaved with those shown in A (ANOVA for repeated measures: F_(1,23)_ = 31.208, p < 0.0001 comparing tetanized slices treated with oTau + DEA/NO vs. oTau). No differences were found between tetanized slices treated with DEA/NO alone vs. DEA/NO + oTau (F_(1,26)_ = 0.052, *p* = 0.821) and DEA/NO alone did not modify potentiation (DEA/NO vs. vehicle: F_(1,22)_ = 0.155, *p* = 0.697). Upper panel: representative traces of fEPSP before (gray) and after tetanus (black) for experiments shown in A. **b** Basal neurotransmission was not affected by oTau, DEA/NO or DEA/NO + oTau in slices that were not tetanized compared with slices treated with vehicle (F_(3,16)_ = 0.950, *p* = 0.440; *n* = 5 slices from 5 animals, 3 males and 2 females for each condition). **c** Quantification of the residual potentiation at 30 and 120 min after tetanus (average of the last 5 min recording from LTP data shown in a and b). Vehicle: *n* = 10 slices/8 animals, 4 males and 4 females; oTau: *n* = 11 slices/8 animals, 4 males and 4 females; DEA/NO: *n* = 14 slices/12 animals, 6 males and 6 females; DEA/NO + oTau: n = 14 slices/11 animals, 6 males and 5 female. One-way ANOVA among all: F_(3,45)_ = 1.124, *p* < 0.0001 at 30 min; F_(3,35)_ = 28.180, p < 0.0001 at 120 min. Bonferroni’s: p < 0.0001 between oTau and other experimental conditions at 30 min and 120 min. The horizontal bars indicate the period during which drugs were added to the bath solution, and arrows indicate tetanus delivery here and in the following figures. ****p < 0.0001

Given that CREB phosphorylation can be enhanced through up-regulation of the NO cascade, we determined whether an increase in NO levels is capable of counteracting the oTau-induced defect in the memory electrophysiological surrogate, LTP [1, 2]. As previously shown [1], LTP was suppressed in hippocampal slices exposed to oTau (100 nM) for 20 min prior to tetanization compared to vehicle-treated slices (Fig. 2a). However, a brief perfusion of 5 min with the NO donor, 2-(*N*,*N*-dethylamino)-diazenolate-2-oxide diethylammonium salt (DEA/NO), at a concentration of 3 μM was sufficient to rescue the oTau-induced LTP suppression (Fig. 2a) without affecting baseline transmission in slices that were not tetanized (Fig. 2b). Moreover, DEA/NO alone did not enhance LTP per se (Fig. 2a), nor affected basal neurotransmission in slices that were not tetanized (Fig. 2b). These slices showed similar levels of potentiation as tetanized slices treated with DEA/NO paired with oTau or slices treated with vehicle both at 30 min and 120 min after tetanus (Fig. 2c). Altogether, these results demonstrate that NO protects against oTau-induced inhibition of LTP.

### sGC is involved in the beneficial effect of NO elevation against oTau-induced impairment of LTP

**Fig. 3:**
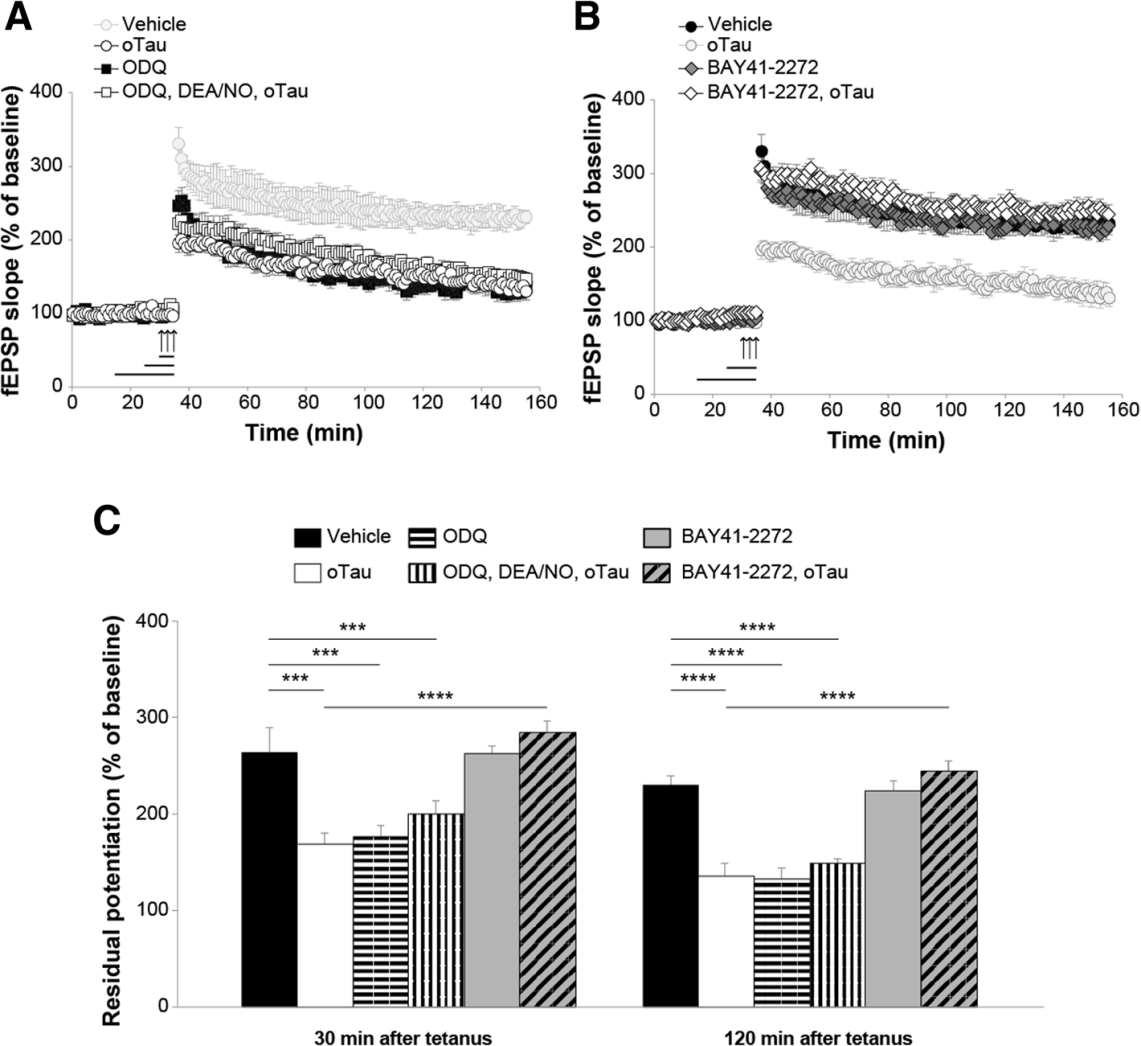
sGC activation protects against the detrimental effect of oTau onto LTP. **a** The sGC inhibitor ODQ (10 μM, 10 min) blocks the rescue of oTau (100 nM, 20 min) induced LTP reduction by DEA/NO (3 μM, 5 min) (ANOVA: F_(1,20)_ = 2.346, *p* = 0.141 comparing ODQ + DEA/NO + oTau vs. ODQ; F_(1,15)_ = 0.004, *p* = 0.954 vs. oTau). The color of the vehicle group is faint because it corresponds to the same group as in B. **b** Perfusion with BAY41–2272 (100 μM, 10 min) rescued the LTP impairment in slices concomitantly treated with oTau (100 nM, 20 min) in experiments interleaved with those shown in A (BAY41–2272 + oTau: n = 11; ANOVA: F_(1,17)_ = 78.187, *p* < 0.0001 comparing BAY41–2272 + oTau vs. oTau). BAY41–2272 alone did not modify potentiation (BAY41–2272 alone: n = 11, vs. vehicle: *n* = 7; F_(1,16)_ = 0.025, *p* = 0.877). No significant differences were found between tetanized slices treated with BAY41–2272 + oTau or BAY41–2272 (F_(1,20)_ = 4.274, *p* = 0.052 comparing BAY41–2272 + oTau with BAY41–2272). The color of the oTau group is faint because it corresponds to the same group as in A. **c** Quantification of the residual potentiation from LTP curves shown in A and B at 30 and 120 min after tetanus. (Vehicle: n = 7 slices/7 animals, 3 males, 4 females; oTau alone *n* = 8 slices/7 animals, 4 males, 3 females; ODQ: *n* = 9 slices/8 animals, 4 males, 4 females; ODQ + DEA/NO + oTau: *n* = 13 slices/11 animals, 6 males, 5 females; BAY41–2272: n = 11 slices/10 animals, 5 males, 5 females; BAY41–2272 + oTau: n = 11 slices/10 animals, 5 males, 5 females). One-way ANOVA: F_(5,53)_ = 14.218, p < 0.0001 at 30 min and F_(5,53)_ = 28.031, p < 0.0001 at 120 min; Bonferroni’s: p < 0.0001 and p < 0.005 between Vehicle and oTau or ODQ or ODQ + DEA/NO + oTau at 30 and 120 min, respectively; p < 0.0001 between oTau and oTau + BAY41–2272 at 30 and 120 min. ***p < 0.005, ****p < 0.0001

NO is a signaling molecule that binds to and stimulates sGC, among other substrates [43]. sGC, in turn, catalyzes the conversion of GTP in cGMP. To investigate whether cGMP production is needed by DEA/NO to rescue oTau-induced impairment of LTP, we treated hippocampal slices with 1*H*-[1, 2, 4] oxadiazolo [4,3-a]quinoxalin-1-one (ODQ), an irreversible inhibitor of NO-sensitive sGC [44]. Perfusion with 10 μM ODQ for 10 min prior to tetanization prevented the protective effect induced by DEA/NO paired with 100 nM oTau for 20 min (Fig. 3a). Moreover, ODQ alone reduced LTP to levels equal to those obtained with oTau without affecting baseline transmission during perfusion (Fig. 3a).

The block of the positive outcome of the administration of DEA/NO against oTau-induced damage of LTP by ODQ is consistent with the interpretation that sGC is in the pathway of the NO beneficial effect. However, alternative explanations are also possible, including the possibility that ODQ might have acted by simply disrupting the physiological mechanisms needed to support LTP. Thus, to directly define whether activation of sGC at the downstream level of NO can be beneficial against oTau-induced damage of synaptic plasticity, in interleaved experiments with those shown in Fig. 3a, we perfused hippocampal slices with the sGC stimulator 3-(4-amino-5-cyclopropylpyrimidine-2-yl)-1-(2-fluorobenzyl)-1H-pyrazolo [3,4-b] pyridine (BAY41–2272), targeting different sGC isoforms without affecting PDE activity [45]. When BAY41–2272 (100 nM, 10 min) was paired with oTau (100 nM, 20 min), LTP reduction caused by oTau was no longer present (Fig. 3b). BAY41–2272 (100 μM) alone, 10 min prior tetanization, did not modify the amount of potentiation, nor affected baseline transmission during perfusion (Fig. 3b). No significant difference was found between tetanized slices treated with BAY41–2272 paired with oTau compared with slices treated with BAY41–2272 alone both at 30 min and 120 min after tetanus (Fig. 3c). Altogether, these findings indicate that stimulation of sGC might play a beneficial role against oTau-induced LTP reduction.

### Elevation of cGMP levels protects against oTau-induced LTP impairment

**Fig. 4:**
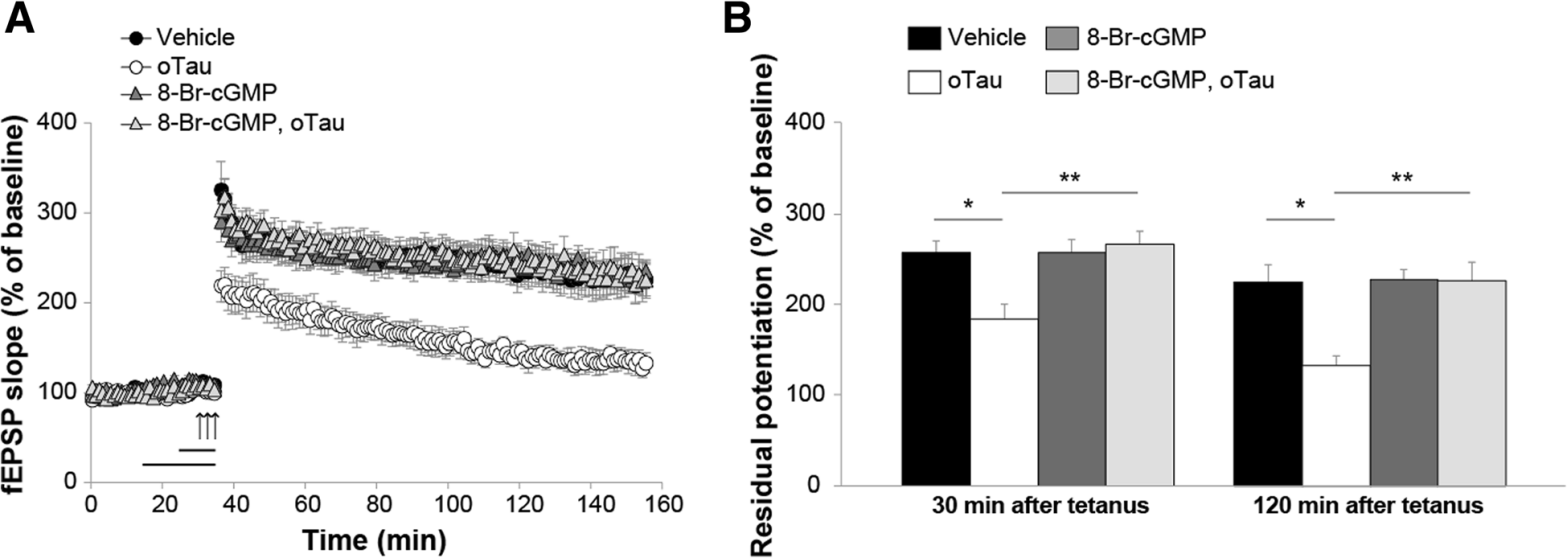
The cGMP analog, 8-Br-cGMP, protects against oTau-induced LTP impairment. **a** Slice perfusion with 8-Br-cGMP (1 μM, 10 min, n = 13) rescued oTau-induced LTP impairment (100 nM, 20 min, n = 8; ANOVA for repeated measures: F_(1,19)_ = 18.537, p < 0.0001). 8-Br-cGMP alone did not modify potentiation (n = 13 vs. 7 in vehicle-treated slices; F_(1,18)_ = 0.001, *p* = 0.974 compared to vehicle). No difference was found between tetanized slices treated with 8-Br-cGMP vs. 8-Br-cGMP + oTau (F_(1, 24)_ = 0.065, *p* = 0.802). **b** Residual potentiation at 30 and 120 min after tetanus from data shown in A. (Vehicle: n = 8 slices/7 animals, 3 males and 4 females; oTau: n = 7 slices/7 animals, 3 males and 4 females; 8-Br-cGMP: 13 slices/11 animals, 6 males and 5 females; 8-Br-cGMP + oTau: 13 slices/11 animals, 5 males and 6 females). One-way ANOVA: F_(3,37)_ = 5.472, *p* = 0.003 at 30 min and F_(3,37)_ = 6.670, *p* = 0.001 at 120 min. Bonferroni’s: *p* = 0.038 and *p* = 0.011 between oTau and vehicle at 30 and 120 min, respectively; p = 0.003 and *p* = 0.002 between oTau and 8-Br-cGMP + oTau at 30 and 120 min, respectively

sGC is known to mediate LTP and memory formation through elevation of intracellular cGMP levels [43]. Thus, we hypothesized that the administration of cGMP analogs might protect against oTau-induced impairment of LTP. We used 8-Br-cGMP, a permeable cGMP analog that specifically activates PKG at low concentrations (*K*_a_, 0.01–0.21 μM for PKG, 12 μM for PKA) [46, 47]. When hippocampal slices were perfused with oTau (100 nM, 20 min) paired with 8-Br-cGMP for 10 min (1 μM) before tetanus, LTP was no longer reduced (Fig. 4a-b). Furthermore, the protection was not caused by an effect of 8-Br-cGMP on LTP, because perfusion with the analog alone did not enhance the amounts of potentiation (Fig. 4a-b). Finally, 8-Br-cGMP alone did not affect baseline transmission during perfusion (Fig. 4a).

**Fig. 5:**
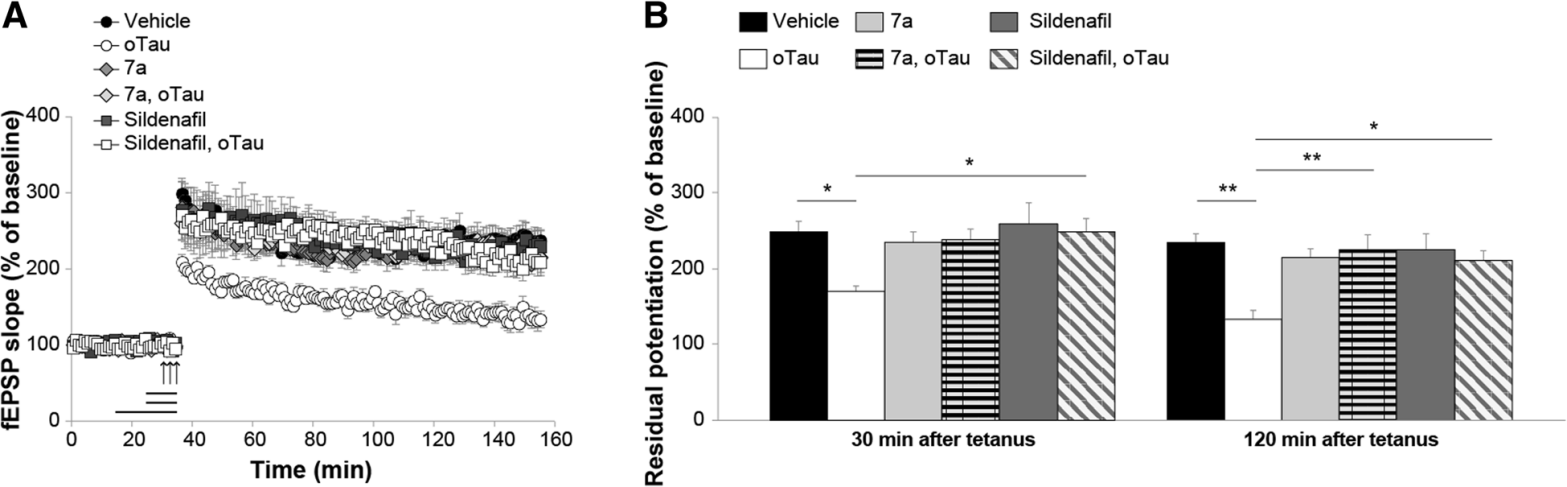
cGMP elevation through PDE5 inhibition protects against oTau-induced LTP impairment. **a** Perfusion with compound 7a (50 nM) or sildenafil (50 nM) for 10 min rescued the LTP impairment in oTau-treated slices (7a + oTau: n = 10; sildenafil + oTau: n = 11; oTau: n = 10; ANOVA for repeated measures: F_(1,18)_ = 20.747, p < 0.0001 and F_(1,19)_ = 34.688, p < 0.0001 compared with oTau-treated slices, respectively). 7a or sildenafil alone did not modify potentiation (7a: n = 11; sildenafil: n = 11; vehicle: n = 9; F_(1,18)_ = 0.156, p = 0.697 and F_(1,18)_ = 0.016, *p* = 0.900). No significant differences were found between tetanized slices treated with 7a vs. 7a + oTau (F_(1,19)_ = 0.060, *p* = 0.809) and between tetanized slices treated with sildenafil vs. sildenafil + oTau (F_(1,20)_ = 0.013, *p* = 0.910). **b** Residual potentiation at 30 and 120 min after tetanus from data shown in C (Vehicle: n = 9 slices/9 animals, 5 males and 4 females; oTau: n = 10 slices/7 animals, 3 males and 4 females; 7a: 11 slices/9 animals, 4 males and 3 females; 7a + oTau: 10 slices/10 animals, 5 males and 5 females; sildenafil: 11 slices/10 animals, 5 males and 5 females; sildenafil + oTau: 11 slices/9 animals, 5 males and 4 females). One-way ANOVA: F_(5,56)_ = 3.44, *p* = 0.009 at 30 min and F_(5,56)_ = 5.673, p < 0.0001 at 120 min. Bonferroni’s: p = 0.04 and p = 0.001 between oTau and vehicle at 30 and 120 min, respectively; p = 0.002 between oTau and 7a + oTau at 120 min; p = 0.03 and p = 0.01 between oTau and sildenafil + oTau at 30 and 120 min, respectively. *p < 0.05, **p < 0.005

cGMP level is maintained through a balance between its production, catalyzed by sGC, and its degradation, catalyzed by PDEs. Therefore, another strategy to increase cGMP level is to use PDEs inhibitors. Specifically, we used two inhibitors of PDE5, sildenafil and compound 7a. Sildenafil is a well-known and extensively studied PDE5 inhibitor with an IC_50_ of 6.0 nM and in vivo half-life of 0.4 h in rodents (~ 4 h in humans) [48, 49]. However, the selectivity ratio for PDE1 and PDE6 is 180 and 12, respectively [50]. Compound 7a possesses higher selectivity for PDE5 (PDE5/PDE6 > 1000) with an IC_50_ of 0.27 nM and plasma half-life of 1.33 h in rodents [35]. We found that a 10 min perfusion with sildenafil (50 nM) in the presence of oTau counteracted the LTP reduction (Fig. 5a-b). Similarly, a 10 min perfusion with 7a (50 nM) rescued the LTP defect in slices concomitantly perfused with oTau (Fig. 5a-b). This phenomenon could not be attributed to an effect of PDE5 inhibition on LTP per se since perfusion with sildenafil or 7a alone did not affect the amount of potentiation, nor to an effect on basal neurotransmission since the two inhibitors did not affect basal synaptic transmission during perfusion (Fig. 5a). Overall, these experiments suggest that elevation of cGMP levels protects against oTau-induced inhibition of LTP.

### PKG activation rescues oTau-induced LTP impairment

**Fig. 6:**
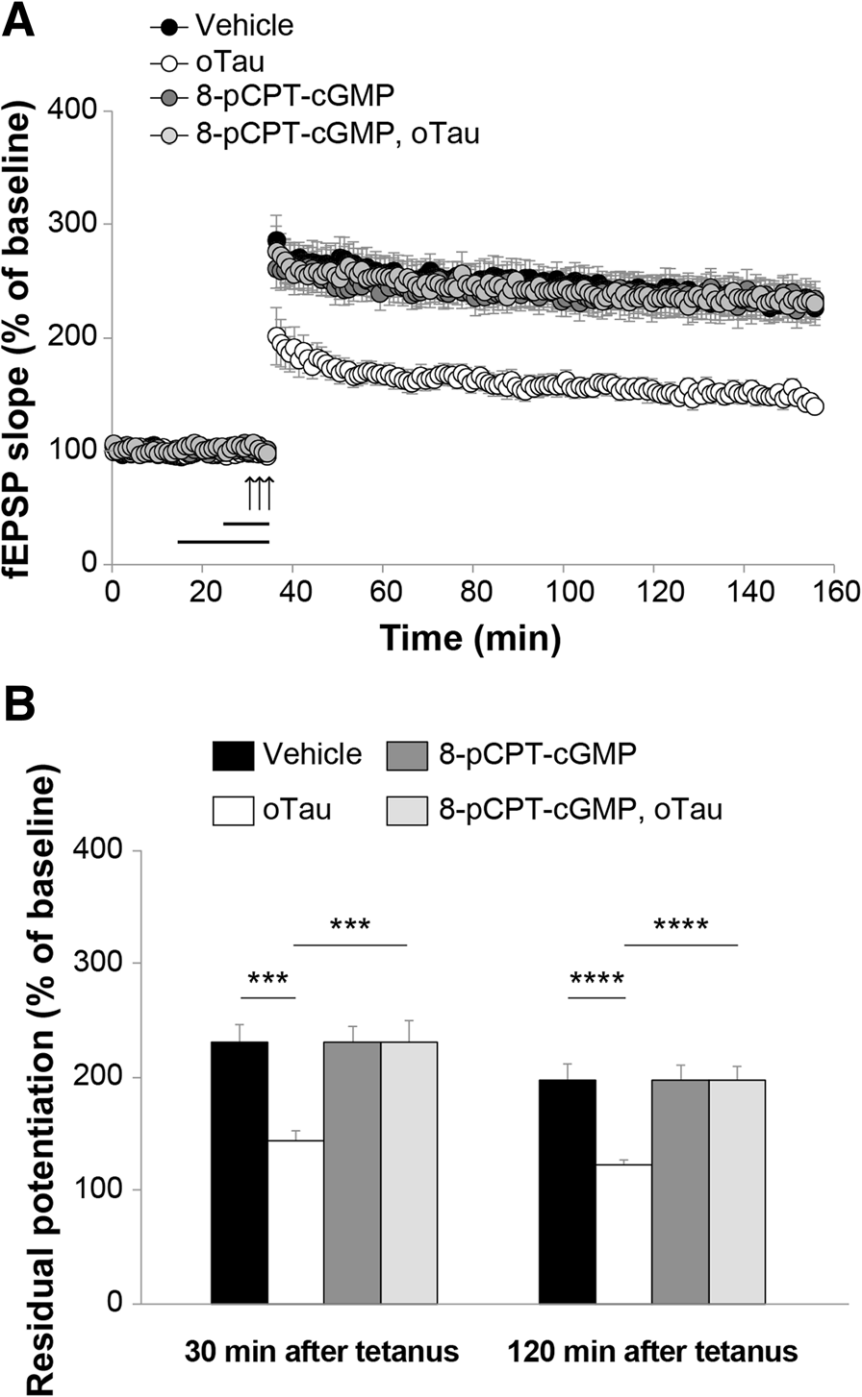
PKG activation counteracts LTP impairment in oTau-treated slices. **a** Perfusion for 10 min with 8-pCPT-cGMP (1 μM) before LTP induction rescued the oTau-induced LTP reduction (n = 10; ANOVA for repeated measures: F_(1,19)_ = 24.037, p < 0.0001 compared with slices perfused with oTau). Perfusion with oTau 20 min prior to tetanization diminished LTP (oTau: n = 10; vehicle: n = 11; F_(1,19)_ = 26.310, *p* = 0.0001 comparing oTau vs. vehicle) while 8-pCPT-cGMP alone did not affect potentiation (n = 11; F_(1,20)_ = 0.100, *p* = 0.755, compared with vehicle). **b** Quantification of the residual potentiation at 30 and 120 min from LTP curves shown in A (Vehicle: n = 11 slices/11 animals, 5 males and 6 females; oTau: n = 10 slices/9 animals, 5 males and 4 females; 8-pCPT-cGMP: 11 slices/10 animals, 5 males and 5 females; 8-pCPT-cGMP + oTau: 10 slices/10 animals, 5 males and 5 females). One-way ANOVA among all: F_(3,38)_ = 8.496, p < 0.0001 at 30 min and F_(3,38)_ = 9.964, p < 0.0001 at 120 min; Bonferroni’s: p = 0.001 and p < 0.0001 between oTau and Vehicle or oTau + 8-pCPT-cGMP at 30 min and 120 min, respectively. *** p < 0.005, ****p < 0.0001

PKG is activated by cGMP. We, therefore, used the specific PKG activator, 8-pCPT-cGMP, to determine whether activation of the kinase protects against oTau-induced impairment of LTP. This compound has higher lipophilicity and membrane permeability than 8-Br-cGMP, and is selective for activation of the two isoforms of PKG, PKGI (K_a_ of 0.05 μM) and PKGII (K_a_ of 0.0035–0.08 μM) compared with other cGMP targets such as PDEs [51]. Initially, we confirmed that 20 min perfusion with 100 nM oTau blocked LTP (Fig. 6a-b). Ten-minute perfusion with 8-pCPT-cGMP (1 μM) before potentiation, in the presence of oTau, abolished LTP suppression. The phenomenon could not be attributed to an effect of PKG activation on LTP per se, since perfusion with 8-pCPT-cGMP alone did not affect the amount of potentiation (Fig. 6a-b), nor to an effect on basal neurotransmission since the activator did not affect basal synaptic transmission during perfusion (Fig. 6a). Altogether, these experiments suggest that PKG activation protects against oTau-induced inhibition of LTP.

### Elevation of cGMP levels and activation of PKG rescue memory impairment in mice injected with oTau

**Fig. 7:**
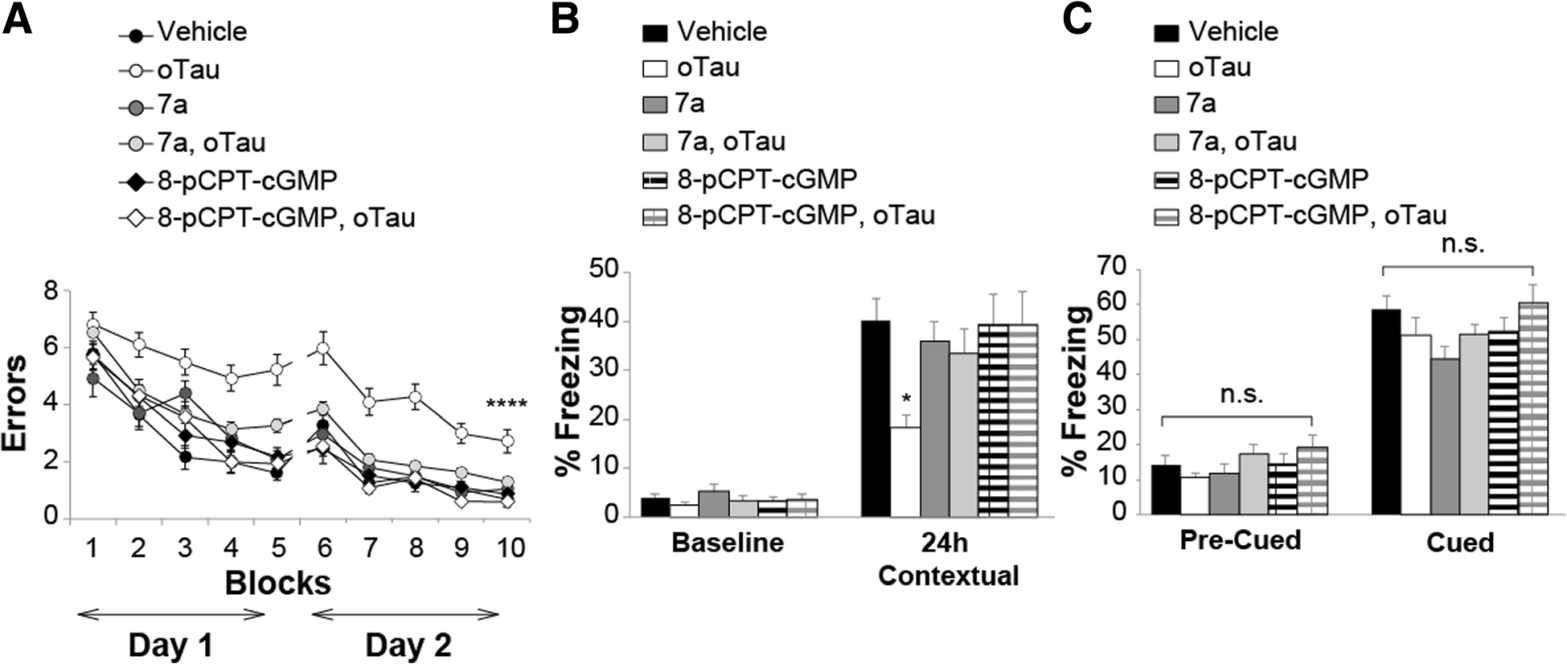
cGMP elevation ameliorates oTau-induced memory impairment. **a** The PDE5 inhibitor 7a (3 mg/kg, i.p.) or the PKG activator 8-pCPT-cGMP (40 μg/Kg, i.p.) protected against the oTau (22.95 μg/ml) induced impairment of RAWM performance). ANOVA for repeated measures among all (day 2): F_(5,83)_ = 17.973, *p* < 0.0001. One-way ANOVA for block 10: F_(5,83)_ = 9.016, p < 0.0001; Bonferroni’s p < 0.0001 oTau vs. vehicle or oTau+ 8-pCPT-cGMP, p = 0.003 vs. oTau+7a. 7a or 8-pCPT-cGMP alone do not modify memory (Bonferroni’s *p* = 1 compared to vehicle, block 10). Vehicle: *n* = 15, 8 males, 7 females, oTau: *n* = 17, 9 males. Eight females, oTau+7a: *n* = 16, 8 males, 8 females, oTau+ 8-pCPT-cGMP: n = 14, 7 males, 7 females, 7a: n = 14, 7 males, 7 females, 8-pCPT-cGMP: n = 13, 6 males, 7 females. **b** 7a (3 mg/kg) or 8-pCPT-cGMP (40 μg/Kg) protected against the oTau-(22.95 μg/ml) induced impairment of contextual memory, without modifying memory per se [24 h: ANOVA F_(5,83)_ = 2.699, p = 0.026; Bonferroni: vehicle vs. oTau: *p* = 0.036; vehicle vs. oTau+7a or oTau+ 8-pCPT-cGMP or 7a or 8-pCPT-cGMP: p = 1]. No differences were detected during baseline assessment (ANOVA among all: F_(5,83)_ = 0.978, *p* = 0.436). Vehicle: n = 16, 8 males, 8 females, oTau: n = 14, 7 males, 7 females, oTau+7a: n = 17, 9 males, 8 females, oTau+ 8-pCPT-cGMP: n = 14, 7 males, 7 females, 7a: n = 15, 8 males, 7 females, 8-pCPT-cGMP: n = 13, 6 males, 7 females. **c** Freezing responses before (Pre) and after (Post) the auditory cue were the same among the six groups shown in B in the cued conditioning test. ANOVA: Pre-cued: F_(5,83)_ = 1.223, *p* = 0.306; Cued: F_(5,83)_ = 2.010, *p* = 0.086

Because the LTP experiments indicate that up-regulation of the NO cascade ameliorates oTau-induced reduction of the memory surrogate LTP, we aimed to extrapolate these findings to memory by using compound 7a and 8-pCPT-cGMP to elevate cGMP levels and activate PKG. At first, we examined spatial working memory through the 2-day radial-arm water maze (RAWM). The task requires short-term reference memory [52]. o-Tau (22.95 μg/ml) was administrated through intra-hippocampal cannulas 180 and 20 min before starting the task. Compound 7a (3 mg/Kg) and 8-pCPT-cGMP (40 μg/Kg) were given i.p. after the 2nd, 4th, 7th and 9th block of trials. We first confirmed that administration of oTau significantly reduced spatial working memory (Fig. 7a). Administration of compound 7a or 8-pCPT-cGMP reversed cognitive decline induced by oTau, since mice performance resembled that of the vehicle-treated mice (Fig. 7a). Additionally, administration of compound 7a or 8-pCPT-cGMP alone did not improve memory performance in vehicle-treated animals (Fig. 7a). Control experiments with the visible platform test excluded that the outcome of these experiments was influenced by an effect on visual, motor and motivational skills since the different groups showed similar swimming speed or time to find the visible platform [see Additional file 1]. Thus, PKG activation is beneficial against oTau-induced impairment of spatial working memory.

Next, we examined the effect of compound 7a and 8-pCPT-cGMP on contextual fear memory. This task depends upon hippocampus and amygdala function [53] and assesses associative memory, a type of memory that is affected in AD patients [54]. We used the same concentration of oTau and drugs as for RAWM experiments. Mice were injected with oTau (180 min and 20 min prior to the training session on the first day), and 7a or 8-pCPT-cGMP immediately after training. There was no significant difference between groups during baseline recording (Fig. 7b). However, as previously demonstrated [1, 37], administration of oTau interfered with memory formation, since mice did not remember the context in which they received the foot shock 24 h after training (Fig. 7b). Administration of compound 7a or 8-pCPT-cGMP rescued contextual memory impairment caused by oTau (Fig. 7b). Compound 7a or 8-pCPT-cGMP did not modify memory per se since freezing did not significantly differ in animals treated with vehicle or compound 7a or 8-pCPT-cGMP alone (Fig. 7b). We also examined cued fear conditioning, which is an amygdala-dependent and hippocampus-independent task [53], without finding differences between groups before or after the cued stimulus (Fig. 7c). Control test showed that different treatments did not change the perception of pain, as determined through sensory threshold assessment (Additional file 1). Finally, administration of oTau, 7a, or 8-pCPT-cGMP did not affect exploratory activity, locomotor function, and anxiety, assessed by the open field test (Additional file 1). Altogether, these findings indicate that upregulation of the NO cascade might ameliorate oTau-induced memory loss.

### Inhibition of PDE5 rescues oTau-induced reduction of pCREB and cGMP levels after memory induction

**Fig. 8:**
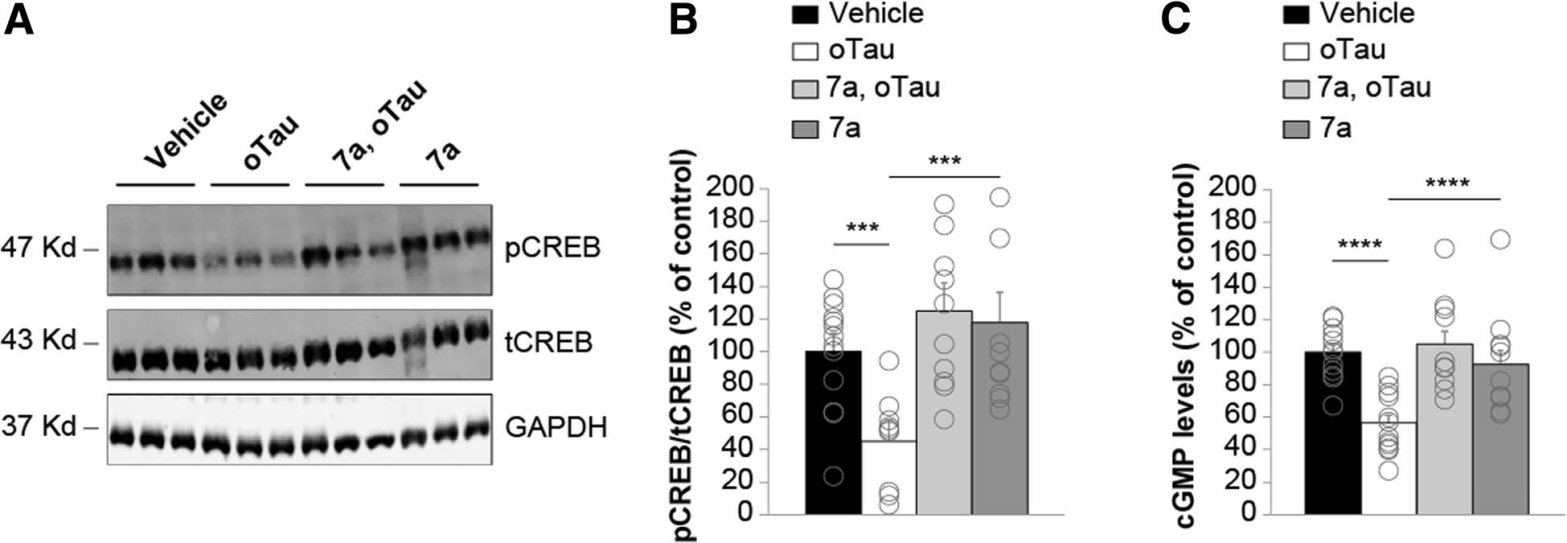
PDE5 inhibition rescues oTau-induced reduction in pCREB and cGMP levels after the electric shock. **a** Immunoblots performed on hippocampi harvested at 1 min after the electric shock. **b** Bar graph shows that oTau significantly decreases pCREB expression, whereas a concomitant treatment with the PDE5 inhibitor 7a is capable of rescuing pCREB expression. The average ratio of p-CREB/t-CREB is displayed (one-way ANOVA: F_(3,45)_ = 6.166; p = 0.001; Bonferroni’s post-hoc: *p* = 0.045 between vehicle and oTau; p = 0.002 between oTau and oTau+7a). GAPDH expression was used as a loading control. Vehicle: n = 14; oTau n = 9; oTau+7a *n* = 12; 7a *n* = 14. **c** Bar graph shows that oTau significantly decreases cGMP level, whereas a concomitant treatment with the PDE5 inhibitor 7a is capable of rescuing cGMP levels (one-way ANOVA: F_(3,41)_ = 9.643; p < 0.0001; Bonferroni’s post-hoc: p < 0.0001 between vehicle and oTau and between oTau and oTau+7a). Vehicle: n = 12; oTau n = 12; oTau+7a n = 10; 7a n = 11

To determine whether the beneficial effects of PDE5 inhibition onto memory formation occurs through rescue of the reduction in pCREB following oTau exposure, we evaluated the effect of elevation of cGMP levels onto pCREB in the presence of the PDE5 inhibitor compound 7a. As shown in Fig. 1, we found that infusion of oTau into dorsal hippocampi (500 nM, 1 μl each side) 180 and 20 min prior to foot-shock reduced pCREB levels (Fig. 8a-b). However, administration of the PDE5 inhibitor 7a (3 mg/kg, i.p. injected 30 min prior to the foot-shock) produced a significant protection against the oTau effect on pCREB levels (Fig. 8a-b).

To further investigate the link between NO cascade and tau-induced impairment in LTP, memory and CREB phosphorylation, we next asked the question whether oTau directly interferes with the cascade by blocking the cGMP increase, which generally occurs immediately after the electric shock [55]. We found that infusion of oTau into dorsal hippocampi (500 nM, 1 μl each side) 180 and 20 min prior to foot-shock reduced cGMP levels (Fig. 8c). However, administration of the PDE5 inhibitor 7a (3 mg/kg, i.p. injected 30 min prior to the foot-shock) rescued the deficit in cGMP levels (Fig. 8c). Altogether, these results indicate that oTau reduces cGMP levels after an electric shock. Furthermore, inhibition of PDE5 can protect against oTau-induced reduction in pCREB and cGMP levels after memory induction.

## Discussion

In the present study, we report the effects of upregulating the NO cascade onto oTau-induced impairment of LTP and memory. The initial observation that inspired this work was the finding that the enhancement of CREB phosphorylation during memory formation was suppressed in animals exposed to oTau. This finding was consistent with the observation that also acetylation levels of H3K27 were reduced by oTau exposure during memory formation. Indeed, CREB protein binds to the histone acetyltransferase CREB-binding protein (CBP) [16, 56], leading to an increase in the acetylation levels of H3 and H4 during spatial and associative learning in the hippocampus and cortex. As numerous studies have demonstrated, phosphorylation of CREB promotes the transcription of memory genes such as *cFos*, *BDNF-IV, EGR1*, *Arc*, and *Nr4a1* and *Nr4a2* that are fundamental in the process of memory formation [57–59]*.* We now corroborate this data by showing that the reduction of CREB phosphorylation in the presence of oTau is associated with reduced expression of *c-Fos* and *Arc* genes. In addition to these observations, a recent study has suggested that tau is a target gene of CREB and negatively regulates its transcription [60]. Moreover, overexpression of CREB significantly reduced mRNA levels of tau by acting on the CRE1 site of the tau promoter to inhibit the transcription of the tau gene [60]. Other studies have demonstrated a similar relationship between pCREB and tau. It was, for example, observed that upregulating the expression of CREB and pCREB attenuates the level of hyperphosphorylated tau in ischemic neurons of the parietal cortex in rat brains [61]. Additionally, it has been shown that the tau/Fyn/NR2B signaling pathway might interfere with CREB activity and expression [62]. Post-translationally modified and hyperphosphorylated tau proteins cause a reduction and a default of activity and phosphorylation of Fyn (tyrosine protein kinase), NR2B (receptor unit of NMDA receptor) and CREB. Impairment of CREB phosphorylation by oTau led us to hypothesize that up-regulation of the NO/cGMP/PKG/CREB pathway that is known to impinge on CREB can be beneficial in AD. To investigate our hypothesis and provide novel insights into the molecular mechanisms underlying oTau-induced defects of learning and memory, we examined the individual components of the NO/cGMP/PKG/CREB signaling pathway in relation to the elevation of oTau levels in mouse hippocampus.

We first investigated the effect of NO on LTP reduction caused by oTau through the NO donor DEA/NO. Our findings suggested that elevation of NO is able to rescue the LTP impairment, providing proof that NO has a protective effect on impaired synaptic strengthening and corroborating other studies showing that NO is involved in hippocampal plasticity processes [26, 63, 64]. Nevertheless, the role of NO has been controversial as several studies have shown that NO is both neuroprotective and neurotoxic. NO has been correlated with neurodegenerative diseases through its formation of reactive nitrogen species [65], but it has correspondingly been shown that NO is able to reduce tau pathology and decrease cell loss, acting as a junction point between Aβ peptides, caspase activation, and tau aggregation [66]. Noticeably, it would depend on where NO is produced that attributes to its role in AD. The neurotoxic NO is produced by microglia inducible NOS (iNOS) which causes synaptic dysfunction through the production of peroxynitrite [67–69]. NO produced by the other isoforms, endothelial NOS (eNOS) and/or neuronal NOS (nNOS) seems to be linked with neuroprotective mechanisms [70]. Interestingly, a recent study has investigated the controversial roles of NO and its effects on synaptic plasticity in 3xTg-AD mice. These animal models often show evidence of dysfunctional calcium-regulated synaptic plasticity before the onset of cognitive deficits occurs, thus suggesting that there must be a compensatory mechanism that allows the hippocampus to maintain its physiological net output, while there is already evidence of synaptic dysfunction. [71]. The work suggested that there is a relationship between the increased calcium release seen in pre-symptomatic AD mice and NO, since NO is calcium-regulated. Block of NO synthesis resulted in a markedly augmented synaptic depression in the AD mice. This data would explain why AD mice and AD patients have elevated nNOS and ryanodine receptor levels [70, 72–74], as a mechanism to boost the NO cascade to compensate for the synaptic dysfunction they experience. At later stages of AD, the cumulative NO levels would reach a level of neurotoxicity and convert the role of the gas molecule from neuroprotective to neurotoxic, demonstrating that NO acts as a Jekyll-Hyde molecule depending on its concentrations [71].

To evaluate the downstream effects of NO, we used the irreversible sGC inhibitor ODQ, which is capable of reducing LTP levels as low as hippocampal slices perfused with tau. Most importantly, pairing oTau with ODQ and DEA/NO blocked the neuroprotective role of the NO donor supporting previous evidence that sGC is an essential feature in the NO signal pathway involved in the rescue of synaptic dysfunction [34, 43]. To exclude the possibility that ODQ might have disrupted some other mechanism involved in the induction of LTP, we used an alternative strategy to investigate the importance of sGC in rescuing oTau-induced synaptic impairment. We utilized the sGC stimulator BAY41–2272 and obtained similar results as those found with DEA/NO, namely BAY41–2272 was able to re-establish normal LTP after exposure to oTau. Interestingly, AD patients were found to have approximately 50% less sGC activity in the superior temporal cortex compared to controls [75]. These observations provide evidence that sGC is highly important for the NO cascade and plays a direct role in the etiopathology of AD. Thus, our findings on the activation of sGC and oTau-induced damage of LTP and memory are consistent with this scenario.

Given that sGC is responsible for producing cGMP from GTP [43], we assumed that increasing cGMP levels would be beneficial and counteract the oTau-induced LTP impairment. In agreement with our hypothesis, both cGMP-analogs 8-Br-cGMP and 8-pCPT-cGMP rescued oTau-induced synaptic impairment to normal physiological levels. Furthermore, we observed that oTau-impaired LTP is restored after perfusion of hippocampal slices with two PDE5 inhibitors, sildenafil and compound 7a, that elevate cGMP levels.

Different PDE enzymes are able to hydrolyze cGMP regulating its intracellular levels. Other groups and we highlighted the importance of PDE5 in modulating the NO-cGMP signal transduction pathway, and thus its effect on synaptic plasticity and memory [35, 36, 76–79]. We previously reported that administration of PDE5 inhibitors sildenafil and compound 7a in a mouse model of amyloid deposition not only increased cGMP levels but also exerted an immediate and long-lasting amelioration of synaptic function and memory [35, 36]. However, a possible point of contention could regard the genuine efficacy of PDE5 inhibitors on improving cognitive aspects, since their effect could have been attributed to the increased blood flow and glucose metabolism (as mentioned above, the initial use of PDE5 inhibitors was for treating hypertension and erectile dysfunction, due to the effect of PDE5 inhibitors on vasodilatation) [80]. Such an argument is unlikely because a previous study showed that the effect of PDE5 inhibition on memory and cognition is unrelated to the cerebrovascular effects [81]. Most importantly, in our in vitro experiments with sildenafil and compound 7a, which exclude the cerebrovascular component, we observed an increased amount of potentiation in slices perfused with oTau. Therefore, the above observations, as well as the design of our study, rule out the possibility that PDE5 inhibitors exert their action through increased vasodilatation.

As with PDE5 inhibitors, the role of PKG in spatial and associative memory has been investigated in oTau-injected mice. In the present study, we demonstrate that intraperitoneal administration of the PKG activator 8-pCPT-cGMP in mice treated with oTau ameliorates memory deficits in the RAWM and fear conditioning tasks. This finding is consistent with the observation that PKG inhibitors block potentiation, and exogenous administration of PKG produces activity-dependent potentiation that mimics the one induced by tetanic stimulation [82]. Accordingly, inhibition of PKG activity post-training for the inhibitory avoidance task prevents memory formation [83]. Thus, we provide evidence that PKG activation ameliorates memory impairment induced by oTau.

Elevation of CREB phosphorylation plays a key role in the induction of synaptic plasticity and memory formation. pCREB increase is known to be present both at 1 min and 60 min after the tetanic stimulation and to induce LTP or memory formation after the electric shock [34, 84–86]. Here we have demonstrated that cGMP elevation rescues the defect in CREB phosphorylation during memory formation and therefore might be therapeutically relevant. Nevertheless, other second messenger cascades impinge on CREB besides the NO pathway and thus might be exploited for drug discovery [16]. Furthermore, cGMP elevation which is known to be transient and is thought to act immediately after LTP or memory induction [34, 55, 87], might have multiple targets in addition to PKG (i.e. the cyclic nucleotide-gated or hyperpolarization-activated cyclic nucleotide-gated ion channels [88]) that might play a role in the rescue of oTau-induced memory loss, in addition to the pathway analyzed in this manuscript. Finally, modulation of downstream targets of PKGs including cytoskeletal organization, vesicle and AMPA receptor trafficking, and gene expression via phosphorylation of various substrates including VASP, RhoA, RGS2, hSERT, GluR1, G-substrate, and DARPP-32 might also be attractive therapeutic targets [88].

To date, there is no clinical evidence suggesting a protective effect of PDE5 inhibitors against AD pathology. A clinical assessment of sildenafil efficacy against memory loss in AD patients would require a chronic administration of the compound to an elderly population. However, probably due to the lack of specificity of the inhibitor that might result in side effects, this has not been attempted. The present study is part of a drug development program aimed at obtaining a highly specific, brain permeant PDE5 inhibitor. Compound 7a has been obtained as a result of this drug discovery effort and its pharmacological efficacy was previously tested in Aβ-induced animal models of AD [35] and is now being assayed in oTau-induced animal models of memory impairment. A relevant finding of this study is the reversal of spatial and associative memory impairment due to intra-cerebral administration of oTau in mice after acute treatment with PDE5 inhibitors. Both types of memory are impaired at early stages of AD [54, 89]. Moreover, an additional investigation was carried out to correlate the enhancement of cGMP levels with the up-regulation of the NO cascade leading to an increase in pCREB levels. An ELISA biochemical assay performed on the hippocampi of mice treated with oTau revealed a dramatic reduction in cGMP elevation during memory formation, which was rescued by the treatment with the PDE5 inhibitor compound 7a. It is noteworthy to mention that the levels of cGMP following induction of LTP could not be measured due to the lack of a quantitative method capable of detecting changes in cGMP levels in the very few fibers located under the stimulating electrode during tetanic stimulation. Nevertheless, the observation that PDE5 inhibition rescues the reduction in cGMP levels after an electric shock strongly suggests that PDE5 inhibition is beneficial against memory loss in oTau pathologies.

Dysregulation of the NO cascade in AD has been shown in proteomic and metabolomic studies [33]. Consistent with this finding, the NO/cGMP/PKG/CREB signaling pathway has been demonstrated to be down-regulated in mouse models of amyloid deposition [34, 36] and its down-regulation has been mainly attributed to the presence of amyloid-beta (Aβ) oligomers [34, 90]. Within the present work, we have extended this observation to soluble forms of tau. Such a parallelism can be due to numerous common features of tau and Aβ oligomers, including beta-sheet structure, aggregation status [1, 91, 92], activity-dependent release [1, 93–95], capability of entering neurons [1, 37, 96–98] and affecting astrocytic intracellular calcium signaling [99–103], and binding to the same cell surface protein, amyloid precursor protein (APP) [104–108].

The discovery of soluble tau aggregates and their involvement in the pathogenesis of AD triggered the development of therapeutics aimed to halt tau aggregation [109–113] or induce tau clearance by immunotherapy [114]. However, concerns have been raised regarding the efficacy of those treatments, since they could reduce the overall NFT load without reducing oTau. Moreover, since NFTs may act as a protective mechanism [115], preventing the spreading of the pathology, the above approaches may be harmful. Additionally, tau plays a crucial role in physiological cell function; thus, therapies directly affecting tau levels might interfere with the physiological role of tau. Immunotherapy is additionally burdened, because most of the antibodies exhibit high affinity for tau, without binding to the specific tau conformers involved in tau seeding [116]. Thereby, we suggest that an alternative strategy to protect against tau-induced memory deficits would be mediated by drugs acting downstream of the NO production. To this regard, the outstanding safety profile of PDE5 inhibitors makes them particularly attractive, as a viable mean to counteract AD.

## Conclusions

**Fig. 9:**
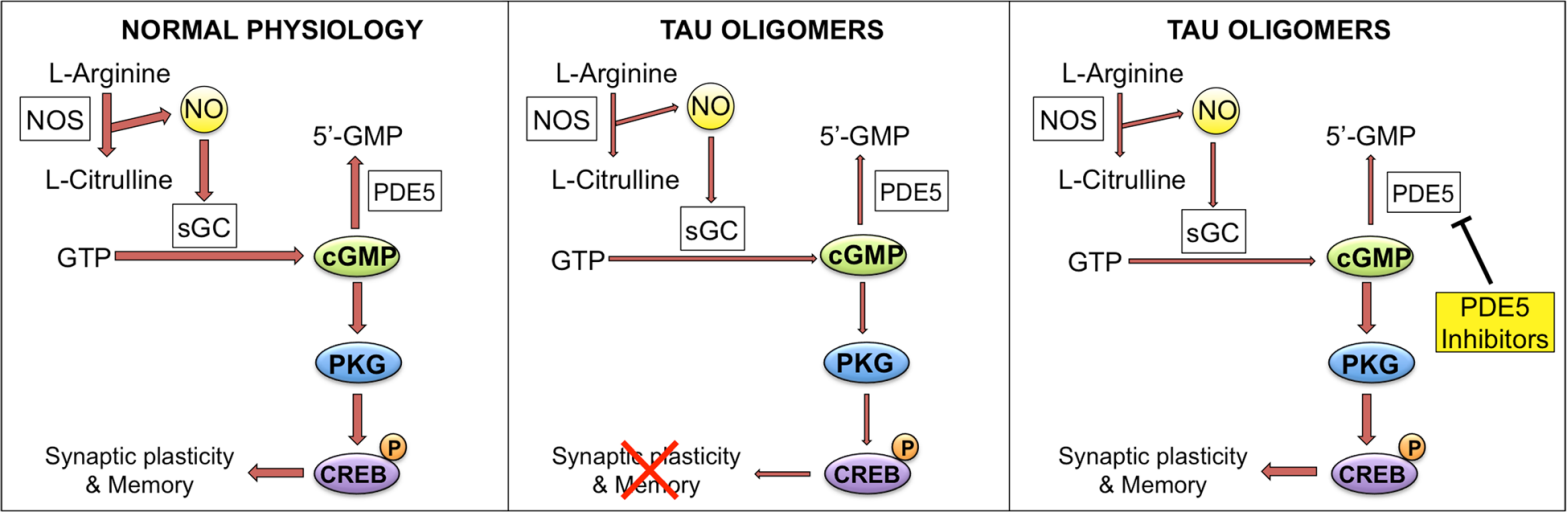
oTau effect on the NO signaling cascade. Left panel shows the cascade under physiological conditions. NO is produced by the enzyme nitric oxide synthase (NOS) that converts L-arginine into L-citrulline. NO activates soluble guanilyl cyclase (sGC), which produces cyclic guanosine monosphosphate (cGMP) from guanosine triphosphate (GTP). cGMP is degraded into 5′-GMP by phosphodiesterase 5 (PDE5). The increase of cGMP levels activates cGMP-dependent protein kinase (PKG), which induces phosphorylation of cAMP-responsive element binding (CREB) and enhancement of synaptic plasticity and memory. In the presence of oTau (central panel), phosphorylation of CREB is reduced as the signaling cascade is down regulated (shown by narrower arrows), leading to an impairment of synaptic plasticity and memory processes. Treatment with PDE5 inhibitors (right panel) rescues the levels of cGMP leading to increased CREB phosphorylation and normal synaptic plasticity and memory

Our findings provide a novel view on how oTau affects synaptic plasticity and memory, pointing at the NO cascade as a second messenger pathway that can be exploited to counteract tau-induced damage of synaptic plasticity and memory (Fig. 9), and offering a new window of therapeutic opportunities against AD and other neurodegenerative diseases characterized by an increase in oTau.

## Additional file

Additional file 1: cGMP elevation does not influence motor, visible or exploratory behavior in mice treated with oTau. Evaluation of speed, latency, sensory threshold and exploratory behavior in mice treated with vehicle, oTau, 7a, 7a + oTau, 8pCPT-cGMP, 8pCPT-cGMP, +oTau. (PDF 171 kb)

## Data Availability

All data generated or analyzed during this study are included in this published article and its supplementary information files.

## Abbreviations

AD: Alzheimer’s disease
APP: Aβ protein precursor
Aβ: Amyloid beta
cGMP: Cyclic guanosine monophosphate
CREB: cAMP-responsive element binding
GTP: Guanosine triphosphate
NFTs: Neurofibrillary tangles
NO: Nitric oxide
NOS: Nitric oxide synthase
oTau: Tau oligomers
pCREB: Phosphorylated CREB
PDE5: Phosphodiesterase 5
PKG: cGMP-dependent protein kinases
RAWM: Radial arm water maze
sGC: Soluble guanylyl cyclase
