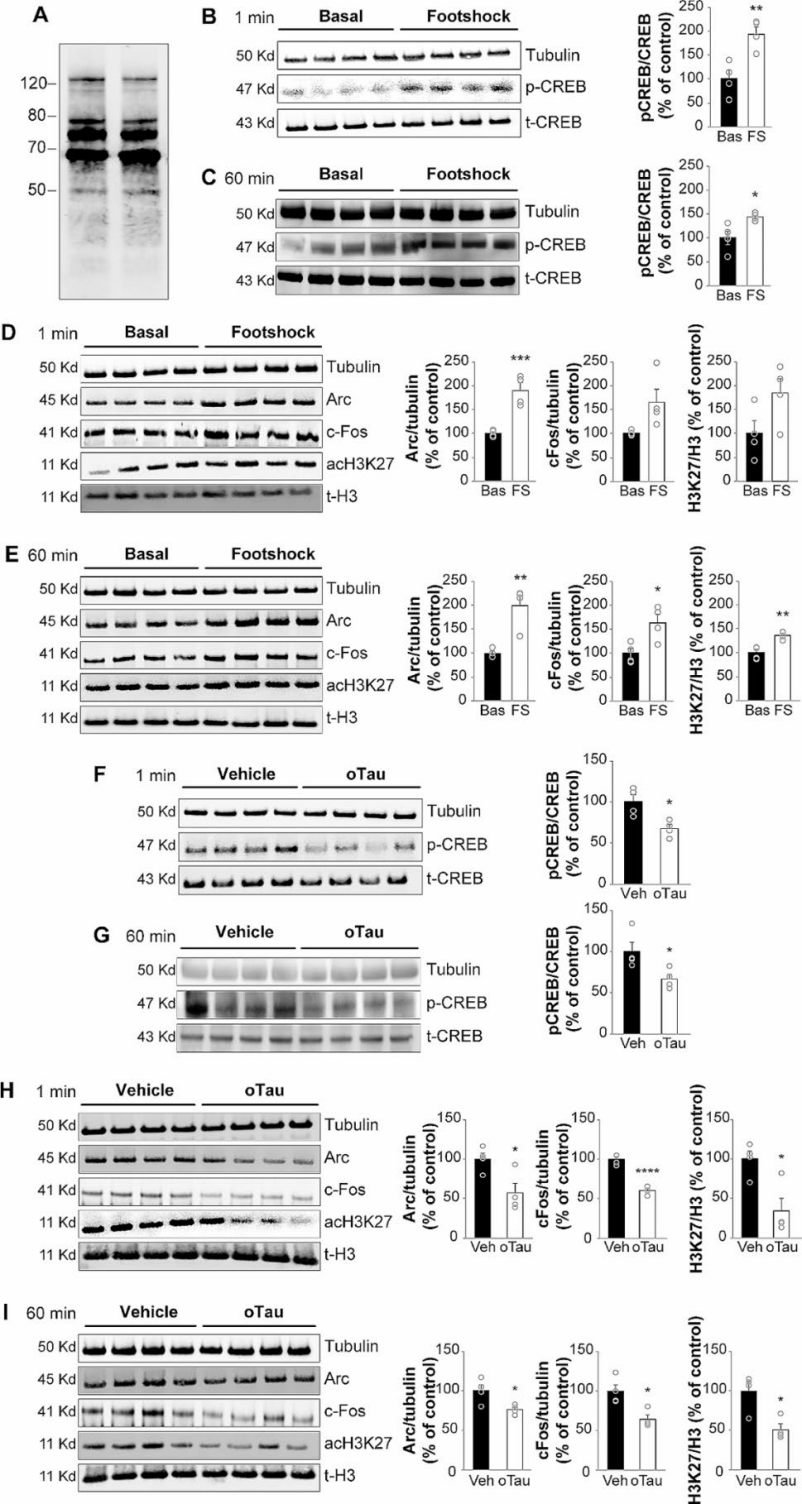# Correction: Synaptic and memory dysfunction induced by tau oligomers is rescued by up-regulation of the nitric oxide cascade

**DOI:** 10.1186/s13024-024-00729-5

**Published:** 2024-04-29

**Authors:** Erica Acquarone, Elentina K. Argyrousi, Manon van den Berg, Walter Gulisano, Mauro Fà, Agnieszka Staniszewski, Elisa Calcagno, Elisa Zuccarello, Luciano D’Adamio, Shi-Xian Deng, Daniela Puzzo, Ottavio Arancio, Jole Fiorito

**Affiliations:** 1 Institute for Research on Alzheimer’s Disease and the Aging Brain, 630 West 168th Street, P&S 12- 420D, New York, NY 10032 USA; 2 DiMi Department of Internal Medicine and Medical Specialties, University of Genoa, Genoa, 16132 Italy; 3 Faculty of Psychology and Neuroscience, Maastricht University, Maastricht, 6229 Netherlands; 4 Department of Biomedical and Biotechnological Sciences, Section of Physiology, University of Catania, Catania, 95125 Italy; 5 Department of Experimental Medicine, Section of General Pathology, School of Medical and Pharmaceutical Sciences, University of Genoa, Genoa, 16132 Italy; 6 Department of Pharmacology, Physiology and Neuroscience, Rutgers University, Newark, NJ USA; 7 Department of Medicine, Columbia University, New York, NY 10032 USA; 8 Oasi Research Institute-IRCCS, Troina, 94018 Italy; 9 Department of Pathology and Cell Biology, Columbia University, New York, NY 10032 USA; 10 Department of Life Sciences, New York Institute of Technology, Northern Boulevard, Theobald Science Center, room 425, P.O. Box 8000, Old Westbury, NY 11568 USA

**Correction: Molecular Neurodegeneration (2019) 14:26**

10.1186/s13024-019-0326-4.

After publication of this work, the authors noted that the tubulin and t-CREB bands in panel B and F were similar. This was due to errors in the panels which likely occurred at the time of assembling the figure during the preparation of the manuscript. After carefully going back to all the raw data and checking the 32 bands assembled in the figure, the authors found and selected exact and correct tubulin and t-CREB bands for both panels, thus correcting the image. The errors only pertain to the incorrect representative images in panels B and F and do not affect any of the analyses or conclusions presented in the paper.