# Supplementary material for: Synaptic and memory dysfunction induced by tau oligomers is rescued by up-regulation of the nitric oxide cascade
Source: Mol Neurodegener. 2019 Jun 27;14:26. doi: 10.1186/s13024-019-0326-4 (PMC6598340; doi:10.1186/s13024-019-0326-4)
Supplement: Supplementary file 1 — cGMP elevation does not influence motor, visible or exploratory behavior in mice treated with oTau. Evaluation of speed, latency, sensory threshold and exploratory behavior in mice treated with vehicle, oTau, 7a, 7a + oTau, 8pCPT-cGMP, 8pCPT-cGMP, +oTau. (PDF 171 kb) [file 13024_2019_326_MOESM1_ESM.pdf]

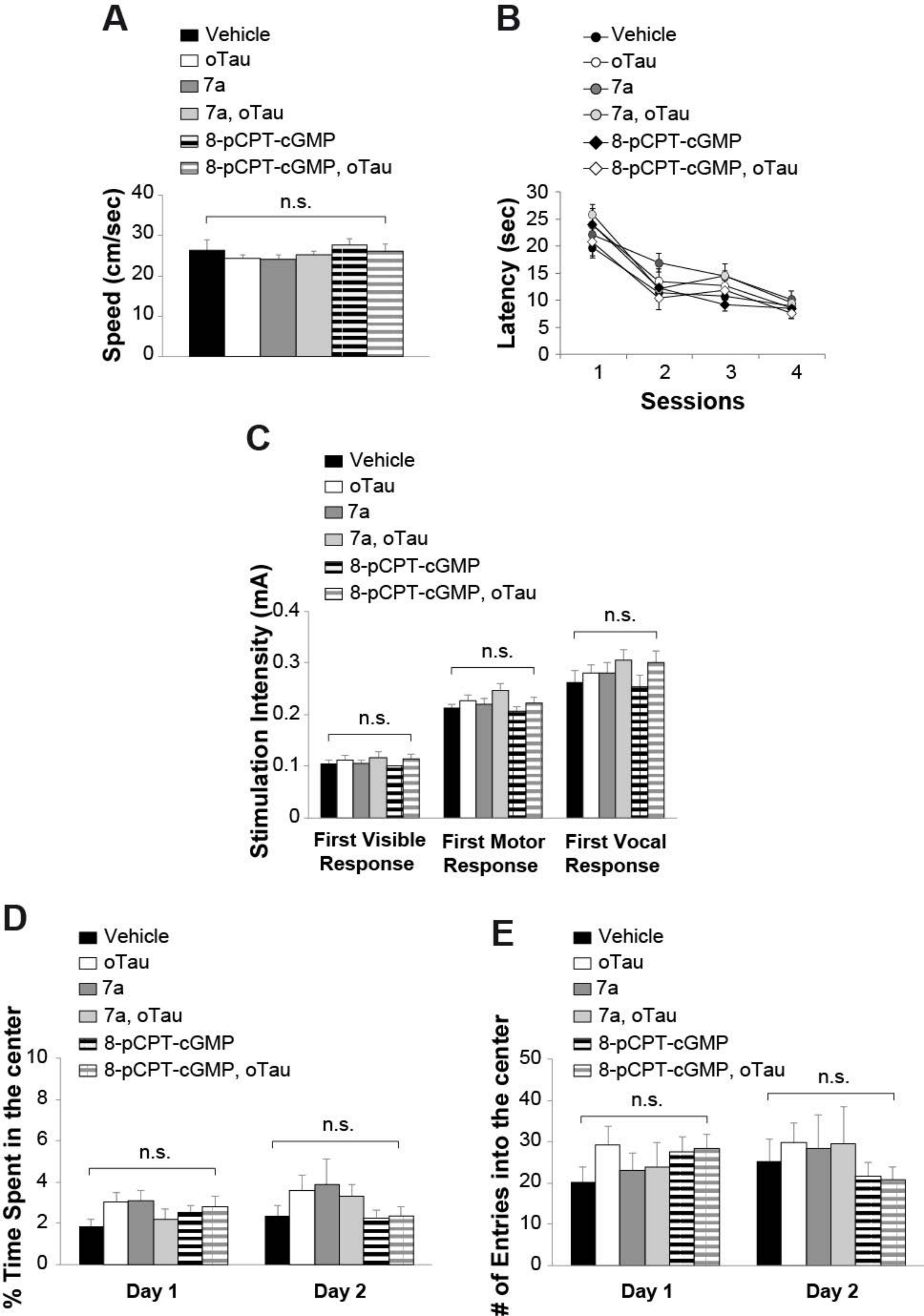

A
Vehicle
oTau
7a
7a, oTau
8-pCPT-cGMP
8-pCPT-cGMP, oTau
Speed (cm/sec)
n.s.
B
Latency (sec)
Sessions
C
Stimulation Intensity (mA)
First Visible Response
First Motor Response
First Vocal Response
D
% Time Spent in the center
Day 1
Day 2
E
# of Entries into the center

Additional File 1 - cGMP elevation does not influence motor, visible or exploratory behavior in mice treated with oTau. A-B) Testing with the visible platform task for assessment of visual-motor-motivational deficits for animals shown in Figure 7A did not reveal any difference in average speed (ANOVA: $F_{(5,83)}$=0.570, p=0.723) (A) and time to reach the visible platform (ANOVA for repeated measures: $F_{(5,83)}$=1.243, p=0.297) (B) among the six groups. Vehicle: n=15, 8 males, 7 females, oTau: n=17, 9 males. 8 females, oTau+7a: n=16, 8 males, 8 females, oTau+8-pCPT-cGMP: n=14, 7 males, 7 females, 7a: n=14, 7 males, 7 females, 8-pCPT-cGMP: n=13, 6 males, 7 females. **C)** No difference was detected among the groups in Figure 7B,C during assessment of the sensory threshold. ANOVA among all: for visible response $F_{(5,83)}$=0.683, p=0.637; for motor response $F_{(5,83)}$=1.756, p=0.131 and for audible response $F_{(5,83)}$=0.933, p=0.464. Vehicle: n=16, 8 males, 8 females, oTau: n=14, 7 males, 7 females, oTau+7a: n=17, 9 males, 8 females, oTau+8-pCPT-cGMP: n=14, 7 males, 7 females, 7a: n=15, 8 males, 7 females, 8-pCPT-cGMP: n=13, 6 males, 7 females. **D-E)** Open field testing showed a similar percentage of time spent in the center compartment ($F_{(5,83)}$=7.037, p=0.407) (D) and the number of entries into the center compartment ($F_{(5,81)}$=0.297, p=0.850) (E) among all conditions at day 2, indicating no differences in exploratory behavior. Vehicle: n=15, 8 males, 7 females, oTau: n=16, 8 males, 8 females, oTau+7a: n=17, 8 males, 9 females, oTau+8-pCPT-cGMP: n=14, 7 males, 7 females, 7a: n=12, 6 males, 6 females, 8-pCPT-cGMP: n=13, 7 males, 6 females.
